# Dr. Charles Wesley Purdy

**Published:** 1901-02

**Authors:** 


Dr. Charles Wesley Purdy, of Chicago, Ill., died in that city January 21, 1901, aged 55 years. He was born in Kingston, Ont., in 1846, and was graduated from Queen's University. Dr. Purdy made a special study of diseases of the kidneys and wrote several books on the subject.

